# A real-time PCR assay with improved specificity for detection and discrimination of all clinically relevant *Bordetella *species by the presence and distribution of three Insertion Sequence elements

**DOI:** 10.1186/1756-0500-4-11

**Published:** 2011-01-21

**Authors:** Lieuwe Roorda, Johannes Buitenwerf, Jacobus M Ossewaarde, Anneke van der Zee

**Affiliations:** 1 Maasstad Laboratory, Molecular Diagnostics Unit, Maasstad Hospital, Olympiaweg 350, 3078HT Rotterdam, The Netherlands; 2 Department of Medical Microbiology and Infectious Diseases, Erasmus MC, Rotterdam, The Netherlands

## Abstract

**Background:**

In Dutch laboratories molecular detection of *B. pertussis *and *B. parapertussis *is commonly based on insertion sequences *IS*481 and *IS*1001, respectively. Both IS elements are more widely spread among *Bordetella *species. Both *Bordetella holmesii*, and *B. bronchiseptica *can harbour *IS*481. Also, *IS*1001 is found among *B. bronchiseptica*. *IS*481, and *IS*1001 based PCR thus lacks specificity when used for detection of specific *Bordetella spp*.

**Findings:**

We designed a PCR based on *IS*1002, another IS element that is present among *Bordetella *species, and exploited it as a template in combination with PCR for *IS*481, and *IS*1001. In combining the PCRs for *IS*481, *IS*1001, and *IS*1002, and including an inhibition control, we were able to detect and discriminate all clinically relevant *Bordetella *species.

**Conclusions:**

We developed an improved PCR method for specific detection of *B. pertussis*, *B. parapertussis, B. holmesii, and B. bronchiseptica.*

## Background

The genus *Bordetella *is comprised of 8 species, 4 of which are known to infect humans; *B. pertussis, B. parapertussis, B. holmesii, *and *B. bronchiseptica. *The most important cause for whooping cough is *B. pertussis*, followed by *B. parapertussis*. *Bordetella holmesii *was first described in 1995 [1], and has since been isolated from patients with a serious underlying disease [2-5]. *B. bronchiseptica *is usually restricted to animals but occasionally is isolated from immunocompromised patients [6,7].

A large number of PCR (polymerase chain reaction) assays have been described for detection of *Bordetella pertussis *and *B. parapertussis*, and more recently *B. holmesii *[8-14]. Most PCRs are based on detection of the insertion sequence elements *IS*481, and *IS*1001, because they exist in multiple copies in the chromosome. A high copy number of PCR target, contributes to the sensitivity of detection. *IS*481 (1053 bp) shows a high degree of homology between different members of *Bordetella *species of 96%. All *IS*1001 (1306 bp) sequences known in Genbank are 100% homologues, as is *IS*1002 (1040 bp). The degree of homology between *IS*481, *IS*1001, and *IS*1002 is less than 5%.

The insertion sequences (IS) that are present in *Bordetella spp*., are distributed according to species and/or host specificity. For example, *IS*1001 is found in all *B. parapertussis*, but *IS*1002 is found only in *B. parapertussis *that infect humans and is absent from *B. parapertussis *sheep isolates [15]. *B. pertussis *harbors both *IS*481 and *IS*1002 while some *B. bronchiseptica *strains may have either *IS*481 or *IS*1001 [15]. *B. holmesii *only has *IS*481.

Due to the distribution of *IS*481, and *IS*1001, PCRs for *B. pertussis *and *B. parapertussis *lack specificity [16]. In recent years many newly developed PCRs were introduced with improved specificity but often with a compromise to sensitivity [11,13,14].

The aim of this study was to improve the specificity of PCR by including another IS element, *IS*1002, as target in PCR. *IS*1002, in addition to *IS*481, and *IS*1001, which enables discrimination of *B. pertussis*, *B. holmesii*, *B. parapertussis*, and *B. bronchiseptica*.

## Methods

*IS*1002 has not been exploited before as a template in PCR detection. We developed a specific *IS*1002 PCR (Table 1) to improve our ability to recognize the correct *Bordetella *species, and to combine it with *IS*481, and *IS*1001 specific PCRs. Addition of Phocine Herpes Virus (PhHV) as internal control acts to monitor the extraction as well as the efficiency of amplification [17].

**Table 1:** DNA sequences of primers and probes directed against three IS elements and the internal control Phocine Herpes Virus (PhHV).

Template	Primer/probe (label)	Sequence (5'-3')
*IS*481	Forward primer	GCCGGATGAACACCCATAAG
	Reverse primer	GCGATCAATTGCTGGACCAT
	Probe (FAM)	CGATTGACCTTCCTACGTC-MGB
*IS*1001	Forward primer	AATTGCTGCAAGCCAACCA
	Reverse primer	CCAGAGCCGTTTGAGTTCGT
	Probe (VIC)	ACATAGACCGTCAGCAG-MGB
*IS*1002	Forward primer	CTAGGTCGAGCCCTTCTTGTTAAC
	Reverse primer	GCGGGCAAGCCACTTGTA
	Probe (CY5)	CATCGTCCAGTTCTGTTGCATCACCC-BBQ
PhHV	Forward primer	GGGCGAATCACAGATTGAATC
	Reverse primer	GCGGTTCCAAACGTACCAA
	Probe (NED)	TTTTTATGTGTCCGCCACCA-MGB

We investigated standard laboratory strains of *Bordetella *(kindly provided by Dr. Frits Mooi and Kees Heuvelman, Laboratory for Vaccine Preventable Diseases, National Institute of Public Health and the Environment, Bilthoven, The Netherlands), which are shown in Table 2. Since detection of *B. pertussis *is of highest concern, we investigated the performance of the newly developed PCR on 100 clinical samples that were previously positive. To verify the specificity of the PCR we investigated 20 clinical respiratory tract samples that were suspect for other pathogens than *Bordetella*. Prior to PCR, laboratory strains were diluted and boiled to release DNA (equivalent to approximately 5 cells/μl). Clinical samples were extracted using EasyMAG (Biomerieux, Grenoble, France).

**Table 2:** Results of PCRs for detection of *IS*481, *IS*1001, and *IS*1002, and the interpretation for identification of clinically relevant *Bordetella *species

*Bordetella *subspecies*	*IS*481	*IS*1001	*IS*1002	identification
*pertussis*	**+**	-	**+**	*B. pertussis*
*parapertussis*	-	**+**	**+**	*B. parapertussis*
*holmesii*	**+**	-	-	*B. holmesii/B. bronchiseptica*
*bronchiseptica*	-	**+**	-	*B. bronchiseptica*
*petrii*	-	-	-	identification not possible
*hinzii*	-	-	-	identification not possible

*Strains were kindly provided by Dr. Frits Mooi and Kees Heuvelman, Laboratory for Vaccine Preventable Diseases, National Institute of Public Health and the Environment, Bilthoven, The Netherlands

PCRs for detection of *IS*481, *IS*1001, and *IS*1002 were performed in a reaction mixture of 25 μl containing 0.5 μM of *IS*481 and *IS*1001 primers, 0.8 μM and 0.6 μM of *IS*1002 Forward and Reverse primer, respectively, and 0.2 μM of PhHV primers. Probes (Table 1) were added with concentrations of 0.14, 0.14, 0.16, and 0.08 μM for respectively *IS*481, *IS*1001, *IS*1002, and the internal control in PCR reaction mix (Sigma -Aldrich (E3004), Munich, Germany). Nine μl of template DNA was added. Amplification was carried out on an ABI 7500 Real-Time PCR system (Applied Biosystems (ABI), Nieuwerkerk a/d IJsel, The Netherlands). The temperature profile included initial denaturation of 4 min. at 94°C, followed by 50 cycles of 94°C for 15 sec., and 60°C for 1 min. Cycle treshold (Ct) values were determined automatically using the ABI SDS software.

## Results

### Evaluation of sensitivity

In order to evaluate the performance of the combination of all four PCRs in a multiplex format performed on standard laboratory strains (Table 2): *IS*481, *IS*1001, *IS*1002, and the internal control, we compared Ct values to each single PCR. No significant differences were found (not shown). Since *IS*481 is present with a much higher copy number in *B. pertussis *than *IS*1002 (approximately 200 and 10 copies, respectively), Ct values of *IS*481 were on average 3.7 lower compared to Ct values of *IS*1002 detection. Of a 10 CFU/ml dilution of *B. pertussis *cells both *IS*481 and *IS*1002 were positive with Ct values of respectively 30.6 and 34.4. The sensitivity of *B. parapertussis *detection was comparable with that using only *IS*1001 as target.

### Evaluation of specificity

To evaluate the specificity of each IS-based PCR, laboratory strains of most known *Bordetella *species (with exception of *B. avium *and *B. trematum*; Table 2), were subjected to the IS-based PCRs. Results were as was expected (Table 2).

To investigate whether all targets were amplified with high fidelity, and would not confound the specificity of detection, we made serial dilutions and subjected these to PCR. With limiting concentrations of *B. pertussis *bacteria, detection of *IS*1002 is lost before detection of *IS*481, because of the difference in copy number. To ensure specificity and to assess a cut-off Ct value for *B. pertussis*, 100 previously positive clinical samples were subjected to PCR. Only when Ct values of *IS*481 >37, *IS*1002 was negative (2% of 100 samples). Thus, a cut-off of Ct 35 should be taken for *IS*481. In 20 respiratory tract samples suspect for other pathogens, no *Bordetella *positives were found. With *B. parapertussis *PCR detection, similar Ct values of *IS*1001 and *IS*1002 were found, and hence no cut-off Ct values are necessary for *IS*1001, and *IS*1002.

### Quality assessment

In addition, the multiplex IS-based PCR was evaluated using the External Quality Assessment Programme of *Bordetella pertussis *from the Quality Control for Molecular Diagnostics (QCMD, Glasgow, UK). The QCMD panel of samples consisted of 5 *B. pertussis *in a range of concentrations, 1 *B. parapertussis*, 1 *B. holmesii*, 2 *B. bronchiseptica*, 1 *B. hinzii*, 1 *Haemophilus influenza*, and 1 negative sample. Using our newly developed PCR we correctly identified *B. pertussis *even in the lowest concentration of 10 CFU/ml, and *B. parapertussis*. *B. holmesii *and one *IS*481 containing *B. bronchiseptica *were recognized correctly and not as *B. pertussis*. *Haemophilus influenza *did not cross react with any of the targeted IS elements.

## Discussion

In this study we included *IS*1002 as target in PCR based detection of *Bordetella*, in addition to *IS*481 and *IS*1001, which is the commonly used PCR in The Netherlands. We aimed to improve the specificity of PCR because the addition of *IS*1002 enables the discrimination of *B. pertussis, B. parapertussis, B. holmesii*, and *B. bronchiseptica*.

In the final report of QCMD (Pierard, D., O. Soetens, and G. Ieven. *Bordetella pertussis *(BPDNA09) EQA Pilot Study. February 2010. QCMD, Glasgow UK.), a high degree of false positivity was observed for detection of *B. pertussis*. It appeared that more than 80% of laboratories contributing to the study used a PCR that is based on *IS*481 and this accounts for the large proportion of false positive results. Indeed, *IS*481 positive samples could either be positive for *B. pertussis*, *B. holmesii*, or *B. bronchiseptica*. Here, we have shown that in our PCR assay these organisms can be discriminated from one another. There are however some limitations. In a previous study [15] it was shown that *IS*481 is very rarely found among *B. bronchiseptica *(1% of strains), in contrast to *IS*1001 that was found in approximately 50% of the studied strain collection. Consequently, approximately 50% of *B. bronchiseptica *carry no known IS elements and thus cannot be detected by IS-based PCR. *B. bronchiseptica *strains that contain a copy of *IS*481 cannot be discriminated from *B. holmesii *using IS-based PCRs. If PCR presents a single *IS*481 positive signal, one might re-investigate the sample using the *B. holmesii *specific PCR targeting the recA gene that was described earlier [18] to discriminate from *B. bronchiseptica*.

The performance of our assay may fail if clinical samples might contain more than one Bordetella species. However, after more than 15 years of experience with PCR detection of *B. pertussis *(*IS*481) and *B. parapertussis *(*IS*1001), we did not find more than one Bordetella species present in clinical specimens.

During the evaluation, we sometimes observed a weak positive signal for *IS*481 (Ct > 37) from *B. hinzii*, although high concentrations of this organism were negative in PCR. This may indicate that *B. hinzii *might contain an *IS*481-like sequence that does not exactly match our PCR. As *B. hinzii *is solely confined to birds, this finding will not confound PCR based on *IS*481, *IS*1001 and *IS*1002 for detection and discrimination of the clinically relevant *Bordetella *species.

## Conclusions

In conclusion, we have developed a real time PCR with improved specificity for detection and discrimination of all clinically relevant *Bordetella *species.

## Competing interests

The author(s) declare that they have no competing interests.

## Authors' contributions

LR carried out the molecular genetic studies, participated in the design of PCR and drafted the manuscript. JB and JMO participated in coordination of the study and helped to draft the manuscript. AZ conceived of the study, and participated in the design. All authors read and approved the final manuscript.
